# Hyperbaric oxygen therapy for radiation enteritis and clinical parameters: a systematic review and meta-analysis

**DOI:** 10.3389/fmed.2025.1632414

**Published:** 2025-10-10

**Authors:** Yuhan Wang, Xuhui Yang, Minjiao Jiang, Zili Tang, Ling Zhao

**Affiliations:** ^1^Acupuncture and Tuina School, Chengdu University of Traditional Chinese Medicine, Chengdu, Sichuan, China; ^2^Acupuncture and Moxibustion College, Nanjing University of Traditional Chinese Medicine, Nanjing, Jiangsu, China; ^3^Acupuncture Clinical Medicine Research Center of Sichuan, Chengdu, Sichuan, China; ^4^Key Laboratory of Acupuncture for Prevention and Treatment of Geriatric Diseases, Ministry of Education, Chengdu, Sichuan, China

**Keywords:** hyperbaric oxygen therapy, radiation enteritis, systematic review, clinical parameters, meta-analysis

## Abstract

**Background:**

Hyperbaric oxygen therapy (HBOT) has been debated regarding its efficacy and safety in treating radiation enteritis (RE), with no standardized clinical protocols established. This study evaluates the clinical evidence on the use of HBOT in the management of RE, focusing on efficacy, safety, treatment parameters, and potential mechanisms.

**Methods:**

A comprehensive computer-based search was conducted across nine databases. The study assessed the effects of HBOT on gastrointestinal symptoms, immune function, inflammation, and its role in reducing the incidence of moderate to severe RE. Additionally, clinical application protocols were analyzed.

**Results:**

This study analyzed data from 22 clinical studies involving 1,318 subjects, including six RCTs incorporated into a meta-analysis. Moderate-quality evidence suggested that HBOT could significantly reduce the incidence of RE [OR = 0.32, 95% CI (0.14, 0.72), *P* = 0.006], particularly showing a significant advantage in decreasing the incidence of grade 3 or higher RE according to the RTOG/EORTC criteria [OR = 0.37, 95% CI (0.17, 0.82), *P* = 0.01]. Additionally, HBOT was shown to effectively improve gastrointestinal symptoms [MD = −1.31, 95% CI (−2.48, −0.13), *P* = 0.03]. Low-quality evidence suggested that HBOT reduced inflammatory markers (IL-6, CRP, TNF-α) and increased immunoglobulin levels (IgA, IgG, IgM). The most commonly reported protocol parameters were 2.0–2.5 ATA, 90–120 min per session, once daily, five times per week, for 30–40 sessions.

**Conclusion:**

This study demonstrates that HBOT effectively alleviates gastrointestinal symptoms, promotes mucosal repair, reduces inflammation, and enhances immune function in patients with RE. HBOT significantly decreases the incidence of RE, particularly severe enteritis. Optimized treatment protocols and individualized adjustments are essential for achieving optimal outcomes. Despite the potential benefits of HBOT for RE, caution is still warranted in clinical practice, with optimization of treatment protocols and individualized adjustments to ensure optimal efficacy.

**Systematic review registration:**

https://www.crd.york.ac.uk/PROSPERO/, CRD42024618971

## 1 Introduction

According to global data from 2020, approximately 19.3 million new cancer cases and nearly 10 million cancer-related deaths were reported across 185 countries, with both incidence and mortality rates continuing to rise (1). Radiotherapy is a core treatment modality for malignant tumors; however, its efficacy is often limited by the collateral damage caused by ionizing radiation to both the targeted areas and the surrounding healthy tissues. Radiation enteritis (RE) is a common and potentially debilitating complication of radiotherapy, affecting approximately 80% of patients undergoing pelvic radiation therapy (2, 3), with 5%–20% of these patients progressing to chronic RE (4). This incidence may still be underestimated (5). The clinical manifestations of RE include hematochezia, increased stool frequency, constipation, mucus in stools, tenesmus, and anal pain. In advanced stages, RE can lead to severe complications such as gastrointestinal bleeding, intestinal perforation, bowel obstruction, and fistula formation. Gastrointestinal symptoms following radiotherapy significantly affect the quality of life in more than half of the patients, with their impact often being moderate to severe (6). Additionally, these symptoms may further contribute to an increased mortality rate in cancer patients (7).

Despite continuous advancements in radiation therapy techniques, the management of radiation enteritis (RE) remains challenging. Conventional treatments, such as anti-inflammatory drugs, antidiarrheal medications, and dietary adjustments, typically provide only limited symptomatic relief and are ineffective in repairing the underlying tissue damage (8, 9). During pelvic radiotherapy, the oral administration of olsalazine or mesalazine has shown limited effectiveness in alleviating acute symptoms. Moreover, olsalazine may exacerbate the incidence and severity of diarrhea (10, 11). The efficacy of antioxidants such as theobromine and the intestinal mucosal protectant glutamine in alleviating symptoms such as abdominal pain and hematochezia remains controversial (12, 13). Patients who undergo argon plasma coagulation (APC) or colostomy may still experience symptoms such as anal pain and tenesmus (8, 14), and may even develop postoperative complications, including intestinal stenosis, rectovaginal fistula, and wound infections (15–17). This gap in treatment underscores the urgent need for effective therapies to alleviate radiation-induced intestinal damage and promote tissue regeneration. Studies show that 83% of patients receiving hyperbaric oxygen therapy (HBOT) experienced objective or complete improvement, with an incremental cost-effectiveness ratio (ICER) of €4,013 per success. Notably, this analysis did not account for additional benefits, such as reduced medication usage, readmission rates, and surgical interventions (18). HBOT demonstrates significant clinical and economic potential in the treatment of radiation-induced injuries, particularly in patients who are unresponsive to conventional therapies. Although multiple studies have shown promising results for HBOT in alleviating symptoms such as pain and hematochezia (19), modulating inflammatory responses (20), and improving endoscopic outcomes in patients with chronic RE (21), one randomized controlled trial (RCT) found no significant difference in efficacy between HBOT and placebo treatment (22). The efficacy of HBOT in the treatment of RE remains controversial, and standardized clinical protocols are currently lacking. Key parameters such as treatment pressure, duration, and frequency, which significantly influence clinical outcomes, have yet to be established. Therefore, this study evaluated the clinical evidence for HBOT in the management of RE, analyzing its efficacy, safety, and treatment parameters, while exploring its potential mechanisms of action. The study seeks to provide support for evidence-based clinical decision-making and offer valuable guidance for optimizing HBOT treatment protocols in the future.

## 2 Materials and methods

### 2.1 Protocol and registration

The study adheres to the PRISMA (Preferred Reporting Items for Systematic Reviews and Meta-Analyses) reporting guidelines. The protocol has been registered with PROSPERO (registration number: CRD42024618971).

### 2.2 Inclusion criteria

#### 2.2.1 Types of studies

This study included randomized controlled trials (RCTs) and non-randomized interventional trials (NRITs). Studies with a minimum of 15 participants were considered.

#### 2.2.2 Population

The inclusion criteria are as follows:

(1)    Patients must meet the diagnostic criteria for cancers, including cervical cancer, prostate cancer, and colorectal cancer, and must have received radiotherapy.
(2)    Tumor diagnostic assessments, including pathological biopsy, endoscopic examination, and imaging studies.
(3)    Diagnosis of RE based on clinical examination.
(4)    Participants aged 18 years or older.
(5)    Patients with other gastrointestinal diseases, such as ulcerative colitis or intestinal tuberculosis, will be excluded.

#### 2.2.3 Intervention and comparison

The intervention group may receive hyperbaric oxygen therapy alone or in combination with routine treatments, whereas the control group will receive conventional treatments, including antioxidants, intestinal mucosal protectants, and anti-inflammatory drugs.

#### 2.2.4 Outcome

The outcome measures include the incidence of RE, the incidence of grade 3 or higher RE according to the RTOG-EORTC evaluation criteria, the LENT-SOMA scale scores, levels of immunoglobulins (IgG, IgM, IgA), and inflammatory markers such as interleukin-6 (IL-6), tumor necrosis factor-alpha (TNF-α), and C-reactive protein (CRP).

### 2.3 Exclusion criteria

The study will exclude duplicate publications, articles without available full text or with incomplete data, systematic reviews, and study protocols.

### 2.4 Data sources and search strategy

The study conducted a literature search across nine databases: China National Knowledge Infrastructure (CNKI), Wanfang Data Knowledge Service Platform (Wanfang), Chinese Scientific Journals Database (VIP), Chinese Biomedical Literature Service System (SinoMed), Wiley, PubMed, Embase, Cochrane Library, and Web of Science. The search period spanned from the inception of each database to November 27, 2024. There were no restrictions on the publication source or language. The MeSH terms used in the search included: [“Hyperbaric oxygen”[MeSH]] AND [“Neoplasm”[MeSH] OR “Cancer” OR “Tumor” OR “Malignancy”] AND [“Radiation enteritis” OR “Radiation colitis” OR “Radiation proctitis”] AND [“Trial” OR “Clinical”]. Additionally, a manual search of the references cited in the identified literature was performed. The detailed list of MeSH terms is provided in the Supplementary File.

### 2.5 Data extraction and management

The study will employ EndNote software for unified management of the literature. Two researchers will independently screen the literature using predefined inclusion and exclusion criteria. They will review titles, abstracts, and full texts to determine whether the studies meet the inclusion criteria. The two researchers will cross-check the literature, and any disputed articles will be referred to a third researcher for final adjudication. Data extraction will be based on information provided in the published articles and Supplementary Materials. If necessary, the original authors will be contacted for additional data. The researchers will independently collect data using a pre-designed data extraction form for subsequent analysis.

### 2.6 Assessment of risk of bias

This study will evaluate the included RCTs using the Cochrane-recommended Risk of Bias assessment tool (23). The assessment covers the following domains: random sequence generation, allocation concealment, blinding of participants and personnel, blinding of outcome assessment, incomplete outcome data, selective reporting, and other potential sources of bias. For NRITs, the Risk of Bias in Non-Randomized Studies of Interventions (ROBINS-I) tool will be used for bias risk assessment. ROBINS-I includes evaluation of biases related to confounding factors, selection of participants into the study, classification of interventions, deviations from intended interventions, missing data, measurement of outcomes, and selection of the reported result (24). Additionally, the study will assess publication bias using funnel plots and Egger’s regression test. If the funnel plot shows asymmetry or if Egger’s test yields a *p*-value < 0.05, the presence of publication bias will be considered (25), and its potential impact on the conclusions will be discussed. Two researchers will independently assess the risk of bias for each included study. In cases of disagreement between the assessors, a third evaluator will be consulted, and the final decision will be made through discussion.

### 2.7 Data synthesis and statistical analysis

This study analyzed data from clinical research. A narrative synthesis was used to analyze the clinical parameters of HBOT, which is suitable for summarizing studies with heterogeneous results (26). For RCTs, meta-analysis was conducted for further assessment. For continuous variable data, mean difference (MD) was used as the effect size; for binary variable data, odds ratio (OR) was used. All effect sizes were reported with 95% confidence intervals (CI). Heterogeneity was assessed using the I^2^ statistic and the *Q*-test. Low heterogeneity was defined as *P* > 0.10 and I^2^ < 50%, and a fixed-effects model was used. High heterogeneity was defined as *P* < 0.10 or I^2^ ≥ 50%, and a random-effects model was applied (27). In the presence of significant heterogeneity, subgroup analysis (e.g., by cancer type, treatment pressure, treatment duration, etc.,) and sensitivity analysis will be conducted, if applicable, to explore potential influencing factors. All analyses were performed using two-tailed tests, with statistical significance set at *p* < 0.05. Results are reported as effect sizes (MD, OR) with 95% confidence intervals (CI). Data analysis was conducted using RevMan 5.4 and Stata 16.0.

### 2.8 Grading of quality of evidence

This study evaluates the quality of evidence using the Grading of Recommendations Assessment, Development, and Evaluation (GRADE) method (28). The GRADE approach classifies evidence quality based on dimensions such as study design, risk of bias, inconsistency, indirectness, imprecision, and publication bias. According to the GRADE method, the final quality of evidence is categorized as follows: (1) High quality: The study results are highly credible and may directly impact clinical practice. (2) Moderate quality: There is some uncertainty in the results, and further research may be needed for confirmation. (3) Low quality: The uncertainty in the results is considerable, and reliable conclusions for clinical decision-making cannot be drawn. (4) Very low quality: The credibility of the study results is very low, and the conclusions may change significantly. Two researchers independently assess the evidence, and any disagreements are resolved through discussion or third-party adjudication.

## 3 Results

### 3.1 Literature search results

**FIGURE 1:**
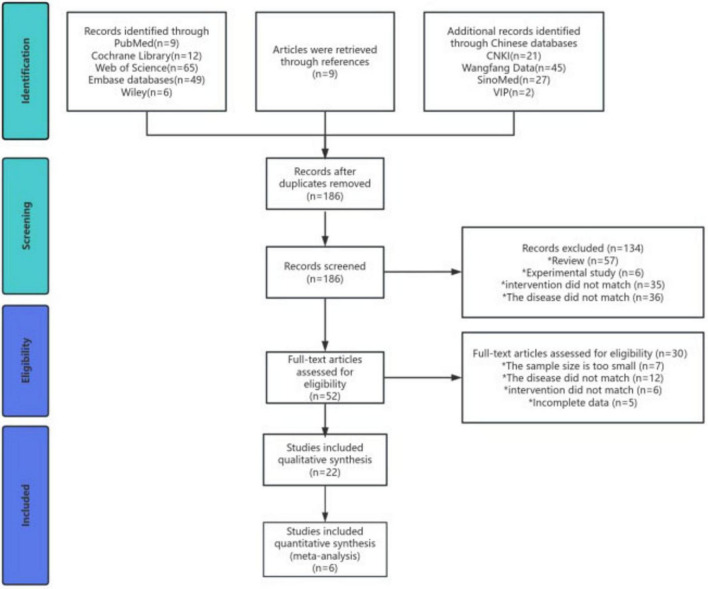
Preferred reporting items for systematic reviews and meta-analyses (PRISMA) flow diagram for study selection.

A total of 245 articles were retrieved in this study. After reviewing the titles, abstracts, and full texts, 22 studies (19–22, 29–46) were finally included [eight RCTs (20–22, 29–31, 33, 34) and 14 NRITs (19, 32, 35–46)]. Among them, six studies (20–22, 29, 31, 33) were included in the meta-analysis, while the remaining studies were included in the qualitative analysis (Figure 1).

### 3.2 The characteristic of clinical studies

The study included 22 clinical trials (19–22, 29–46) with a total of 1,318 participants. The smallest clinical study included 17 participants, while the largest included 120 participants. Of the included studies, seven (20, 21, 29–31, 37, 46) were conducted in China, four (34, 36, 44, 45), in the United States, two (35, 40) in Australia, and two (19, 43) in Portugal, with the remaining seven studies (22, 32, 33, 38, 39, 41, 42) originating from Mexico, the UK, Indonesia, Austria, Sweden, France, and Norway. The types of cancer studied included cervical cancer, prostate cancer, colorectal cancer, and others. Thirteen (21, 22, 31, 32, 34–36, 38–41, 44, 45) clinical trials reported adverse events related to HBOT. Detailed information is provided in Table 1.

**TABLE 1:** Characteristics of the included studies.

Author	Country	Study type	Tumor type	Sample size		Age (year)		Radiation does (Gy)	Intervention measures		Treatment course (week)	Adverse events
				T (F/M)	C (F/M)	T	C		T	C		
Zhu et al. (20)	China	RCT	Cervical cancer	46	46	50.13 ± 8.27	50.93 ± 8.39	Treatment group: external radiation: 53.77 ± 3.68/brachytherapy: 13.14 ± 5.33 Control group: external radiation: 54.19 ± 3.61/brachytherapy: 12.98 ± 5.29	HBOT + RT	RT	5 (2–8)	NA
Wu et al. (29)	China	RCT	Cervical cancer	40	40	51.9 ± 8.9	53.1 ± 9.2	Treatment group: external radiation: 50.8 ± 7.1/brachytherapy: 12.2 ± 1.9 Control group: external radiation: 51.7 ± 8.1/brachytherapy: 12.8 ± 1.7	HBOT + RT	RT	4	NA
Wu et al. (30)	China	RCT	Cervical cancer	32	32	52.6 ± 9.1	54 ± 8.9	Treatment group: external radiation: 52.8 ± 7.2/brachytherapy: 12.9 ± 2.5 Control group: external radiation: 52.4 ± 7.1/brachytherapy: 13.1 ± 2.6	HBOT + moxibustion	HBOT + drug enema	8 (6–10)	NA
Fu et al. (31)	China	RCT	Pelvic tumors	43 (20/23)	43 (18/25)	/	/	Treatment group (range): 40–70 Gy Control group (range): 40–80 Gy	HBOT + RT	RT	8	In the HBOT group, there were 5 cases of headache, 7 cases of nausea and vomiting, and 2 cases of tinnitus.
Glover et al. (22)	UK	RCT	Pelvic tumors	55 (23/32)	29 (14/15)	62 ± 11	62.3 ± 11	NA	HBOT + RT	sHBOT + RT	8	The most commonly reported adverse events were changes in refractive error, including myopia (3 out of 28 patients in the control group vs. 16 out of 53 patients in the treatment group), fatigue or increased fatigue (3 vs. 2), and ear pain or barotrauma (6 vs. 15).
Sidik et al. (33)	Indonesia	RCT	Cervical cancer	32	33	47 ± 5.5	44.7 ± 6.2	NA	HBOT + RT	RT	/	NA
Hu et al. (21)	China	RCT	Cervical cancer, colorectal cancer	23 (3/20)	20 (2/18)	52.65 ± 8.76	54.29 ± 7.87	NA	HBOT + RT	RT	4	In the HBOT group, 2 patients experienced mild dizziness.
Clarke et al. (34)	USA	RCT	Pelvic tumors	/	/	/	/	NA	HBOT + RT	sHBOT + RT	6	Consistent with hyperbaric oxygen practices, ear pain/ear discomfort (barotrauma) was the most common complaint. Nineteen patients complained of ear pain or discomfort. Otoscopic examination revealed no abnormalities in 11 patients, while 7 patients showed tympanic membrane changes consistent with barotrauma, and 1 patient had both tympanic membrane damage and middle ear effusion. One patient complained of sinus barotrauma, and four patients experienced transient myopia. Two patients complained of claustrophobia.
Woo et al. (35)	Australia	Non-RCT	Pelvic tumors	18 (17/1)	/	/	/	NA	HBOT	/	4 (2–7)	One patient experienced angina, one patient had ear discomfort, and five patients experienced transient blindness.
Marshall et al. (36)	USA	Non-RCT	Pelvic tumors	65 (37/28)	/	/	/	NA	HBOT	/	/	Eleven patients developed temporary oxygen-induced myopia, four patients required the placement of tympanostomy tubes, and one patient experienced a tonic-clonic seizure.
Li et al. (37)	China	Non-RCT	Cervical cancer	24 (10/14)	28	43.2 ± 14.6	/	External radiation (range): 60–70 Gy Brachytherapy (range): 20–30 Gy	HBOT + RT	RT	/	NA
Alvaro-Villegas et al. (32)	Mexico	Non-RCT	Cervical cancer	17	14	51.9 ± 12.5	59.3 ± 12	Treatment group: 78.5 ± 13.9 Gy Control group: 81.1 ± 9.0 Gy	HBOT	APC	/	In the APC group, 5 patients developed complications: 2 patients experienced rectal pain after the first treatment, and 3 patients developed APC-related rectal ulcers at the end of the follow-up.
Moreira Monteiro et al. (19)	Portugal	Non-RCT	Pelvic tumors	88 (73/15)	/	/	/	NA	HBOT	/	12	Eighteen patients experienced barotrauma of the middle ear, and one patient reported reversible myopia.
Mayer et al. (38)	Austria	Non-RCT	Prostate cancer	18	/	/	/	External radiation 50.4/1.8 Gy	HBOT	/	4 (3–8)	No adverse reactions were observed with HBOT.
Oscarsson et al. (39)	Sweden	Non-RCT	Cervical cancer, rectal cancer, prostate cancer	39 (35/4)	/	71 (35–84)	/	Prostate (range): external radiation 70–75 Gy/brachytherapy 28–33 Gy/external radiation 50 Gy + brachytherapy 20 Gy Rectal (range): external radiation 25–50 Gy Cervix (range): external radiation 60–64 Gy	HBOT	/	7 (6–8)	No adverse reactions were observed with HBOT.
Andren and Bennett (40)	Australia	Non-RCT	Pelvic tumors	52 (44/8)	/	67.9 ± 10.1	/	NA	HBOT	/	6 (4–10)	Thirteen patients complained of ear pain during HBOT treatment, with 2 of them having clinically confirmed barotrauma during examination. Four patients complained of myopia.
Ouaïssi et al. (41)	France	Non-RCT	Pelvic tumors	44 (26/18)	/	/	/	NA	HBOT	/	7 (1–18)	Four patients experienced temporary hearing issues, and one patient had transient myopia.
Velure et al. (42)	Norway	Non-RCT	Pelvic tumors	95 (50/45)	/	65 ± 11.6	/	External radio-therapy (range): 35–100 Gy Brachytherapy (range): 7–75 Gay	HBOT	/	6	NA
Gaio-Lima et al. (43)	Portugal	Non-RCT	Pelvic tumors	22 (6/16)	/	64 (40–81)	/	NA	HBOT	/	10 (4–14)	NA
Dall’Era et al. (44)	USA	Non-RCT	Prostate cancer	27	/	/	/	Monotherapy (median): external radio-therapy 68 Gy/brachytherapy 144 Gy Combined therapy (range): external radio-therapy 40–50 Gy + brachytherapy 90–100 Gy	HBOT	/	7 (6–12)	Three patients required the placement of pressure-equalizing tympanostomy tubes, two patients developed transient oxygen-induced myopia, and one patient experienced a seizure.
Hampson et al. (45)	USA	Non-RCT	/	73	/	/	/	NA	HBOT	/	9 (5–12)	Some patients developed myopia, claustrophobia, and tinnitus, and two patients experienced seizures.
Zhang et al. (46)	China	Non-RCT	Pelvic tumors	30 (17/13)	30 (16/14)	62.83 ± 9.59	62.83 ± 9.59	Cervical cancer 50 55 Gy Pancreatic cancer/liver cancer 50 60 Gy Rectal cancer 55 70 Gy	HBOT + RT	RT	4	NA

T, treatment group; C, control group; HBOT, hyperbaric oxygen therapy; sHBOT, sham hyperbaric oxygen therapy; RT, routine treatment; APC, argon plasma coagulation; NA, no available; Gy, gray.

### 3.3 Risk of bias assessment

**FIGURE 2:**
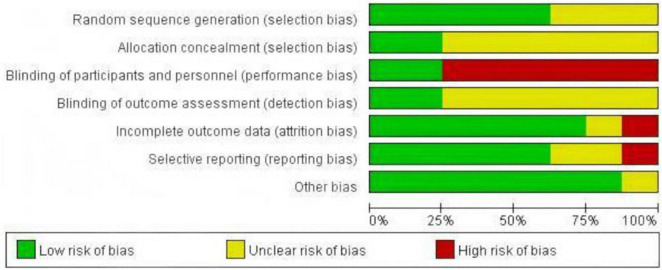
Risk of bias graph.

**FIGURE 3:**
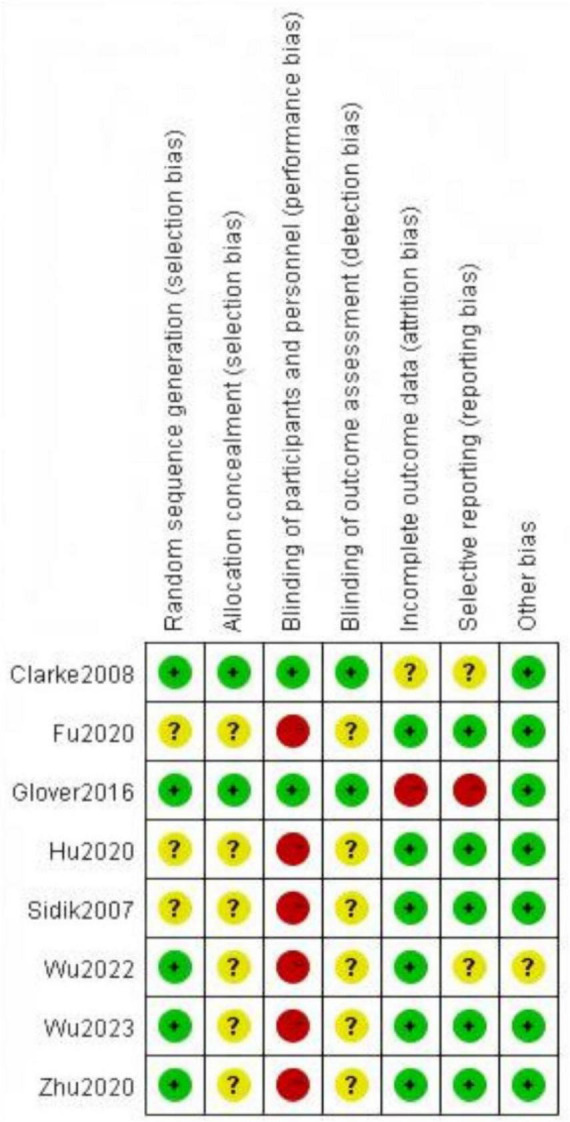
Risk of bias summary.

We assessed the risk of bias for the eight included randomized controlled trials. The results showed that three studies (20, 29, 30) used a random number table, and two studies (22, 34) used computer-generated central randomization, hence these were considered to be at low risk of bias. The remaining three studies (21, 31, 33), which did not specify the randomization method, were classified as having an unclear risk of bias. Two studies (22, 34) provided information on allocation concealment. Six studies (20, 21, 29–31, 33) did not use sham HBOT in the control group, and as a result, blinding of patients and implementers may not have been applied, leading to a high risk of bias. Only two studies (22, 34) reported blinding of outcome assessors. One study (22) did not fully report the trial outcomes, which led us to consider it at high risk for bias related to data integrity and reporting bias. As shown in Figures 2, 3.

The risk of bias assessment for the 14 NRITs revealed that nine (35, 36, 38–41, 43–45) studies had a critical high risk of bias, one study (19) had a serious risk of bias, two studies (32, 46) had a moderate risk of bias, and two studies (37, 42) were judged to have a low risk of bias. Most studies did not adjust for important confounding factors such as age, gender, and extent of tissue damage, and only performed univariate analysis, which resulted in a high risk of confounding bias. Some studies had inconsistent intervention and follow-up durations among participants, which could lead to a high risk of selection bias. Additionally, there were varying degrees of bias risk in areas such as intervention classification bias, deviation from intended interventions bias, missing data bias, and outcome measurement bias. Specific reasons for bias are documented in Supplementary File.

### 3.4 Meta-analysis of overall effect

#### 3.4.1 Incidence of radiation enteritis

**FIGURE 4:**
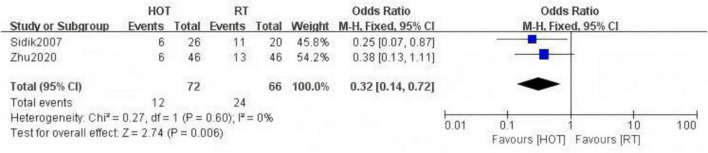
A Forest plot for the incidence of radiation enteritis.

**FIGURE 5:**
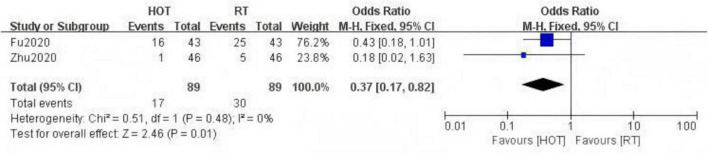
A Forest plot for the incidence of severe radiation enteritis.

The study included two RCTs (20, 33) with a total of 138 participants. The results indicated that HBOT significantly reduced the incidence of RE [OR = 0.32, 95% CI (0.14, 0.72), *P* = 0.006, I^2^ = 0%] (Figure 4). Notably, compared to conventional treatment, HBOT demonstrated a significant advantage in reducing the incidence of RE at RTOG/EORTC grade 3 or higher [OR = 0.37, 95% CI (0.17, 0.82), *P* = 0.01, I^2^ = 0%] (Figure 5).

#### 3.4.2 LENT-SOMA scale

**FIGURE 6:**
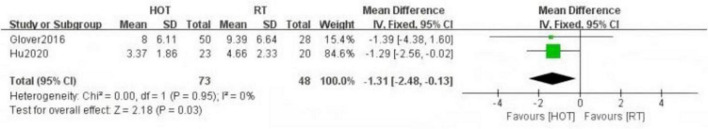
A Forest plot for the LENT-SOMA scale.

Two RCTs (21, 22) utilized the LENT-SOMA scale to evaluate the effect of HBOT on intestinal symptoms in patients with RE. The results showed that, compared to conventional treatment, HBOT significantly reduced the LENT-SOMA score and promoted improvement in intestinal symptoms [MD = −1.31, 95% CI (−2.48, −0.13), *P* = 0.03, I^2^ = 0%] (Figure 6).

#### 3.4.3 Inflammatory markers

**FIGURE 7:**
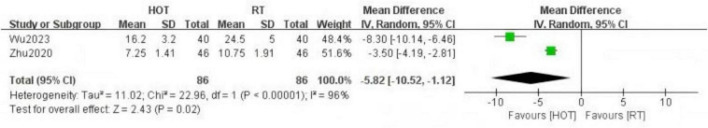
A Forest plot for the inflammatory factor IL-6 (ng/L).

**FIGURE 8:**
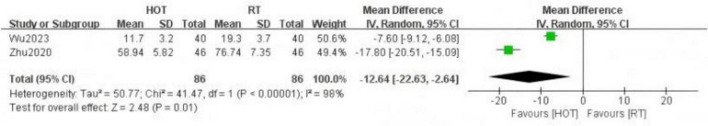
A Forest plot for the inflammatory factor CRP (mg/L).

**FIGURE 9:**
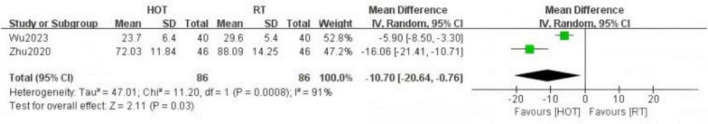
A Forest plot for the inflammatory factor TNF-α (ng/L).

Two studies (20, 29) evaluated the impact of HBOT on inflammatory markers in patients with RE. The results showed that HBOT significantly reduced the levels of inflammatory factors IL-6 (ng/L) [MD = −5.82, 95% CI (− 10.52, −1.12), *P* = 0.02, I^2^ = 96%] (Figure 7), CRP (mg/L) [MD = −12.64, 95% CI (−22.63, −2.64), *P* = 0.01, I^2^ = 98%] (Figure 8), and TNF-α (ng/L) [MD = −10.70, 95% CI (−20.64, −0.76), *P* = 0.03, I^2^ = 91%] (Figure 9).

#### 3.4.4 Immunoglobulin

**FIGURE 10:**
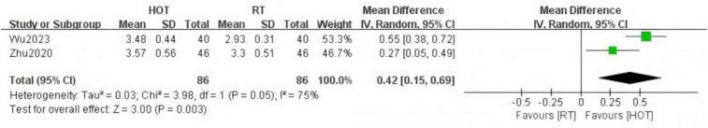
A Forest plot for the immunoglobulin IgA (U/ml).

**FIGURE 11:**
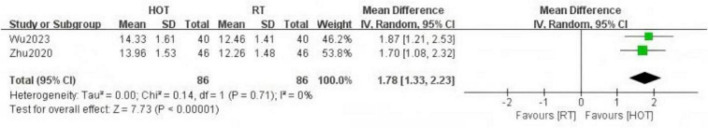
A Forest plot for the immunoglobulin IgG (U/ml).

**FIGURE 12:**
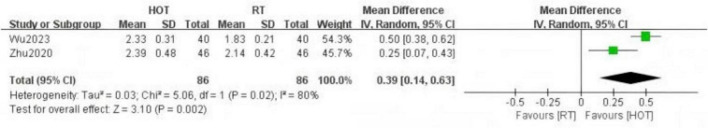
A Forest plot for the immunoglobulin IgM (U/ml).

The study demonstrated that HBOT significantly increased the levels of immunoglobulins IgA (U/ml) [MD = 0.42, 95% CI (0.15, 0.69), *P* = 0.003, I^2^ = 75%] (Figure 10), IgG (U/ml) [MD = 1.78, 95% CI (1.33, 2.23), *P* < 0.00001, I^2^ = 0%] (Figure 11), and IgM (U/ml) [MD = 0.39, 95% CI (0.14, 0.63), *P* = 0.002, I^2^ = 80%] (Figure 12).

### 3.5 Quality of evidence

After assessing the quality of evidence for the study results, it was determined that the incidence of RE, the incidence of RE at RTOG/EORTC grade 3 or higher, and the LENT-SOMA scale were rated as moderate quality. In contrast, the evidence for inflammatory factors and immunoglobulins was rated as low quality. For specific details regarding the quality assessment, please refer to the Supplementary File.

### 3.6 Publication bias

Due to the small number of included RCTs (less than 10), publication bias analysis was not performed.

### 3.7 Clinical parameters of hyperbaric oxygen

The specific parameters of HBOT are provided in Table 2.

**TABLE 2:** Clinical parameters of hyperbaric oxygen therapy.

Author	Atmospheric pressure	Duration of pure oxygen inhalation	Duration of intermittent cabin air inhalation	Treatment frequency		Total duration of each treatment	Total number of hyperbaric oxygen treatments	Treatment course (weeks)	Rate of adherence
Zhu et al. (20)	2 ATA	40 min	10 min	7 times per week	1 time per day	95 min	32 (14–56)	5 (2–8)	NA
Wu et al. (29)	2 ATA	40 min	10 min	5 times per week	1 time per day	100 min	20	4	100%
Wu et al. (30)	2.2 ATA	40 min	10 min	5–7 times per week	1 time per day	95 min	40 (30–50)	8 (6–10)	NA
Fu et al. (31)	2.4 ATA	90 min	10 min	5 times per week	1 time per day	120 min	40	8	NA
Alvaro-Villegas et al. (32)	2–2.5 ATA	NA	NA	NA	1 time per day	90 min	30	NA	NA
Glover et al. (22)	2.4 ATA	90 min	10 min	5 times per week	1 time per day	100 min	40	8	92.38%
Sidik et al. (33)	NA	NA	NA	NA	NA	NA	NA	NA	NA
Clarke et al. (34)	2.0 ATA	NA	NA	5 times per week	1 time per day	90 min	30	6	NA
Hu et al. (21)	2 ATA	60 min	10 min	5 times per week	1 time per day	110 min	20	4	NA
Li et al. (37)	2.4 ATA	90 min	10 min	5–7 times per week	1 time per day	120 min	NA	NA	NA
Moreira Monteiro et al. (19)	2.5 ATA	70 min	5 min	5 times per week	1 time per day	100 min	60	12	NA
Mayer et al. (38)	2.2–2.4 ATA	60 min	NA	7 times per week	1 time per day	NA	26 (20–60)	4 (3–8)	93.06%
Dall’Era et al. (44)	2.4 ATA	NA	NA	5–7 times per week	1 time per day	90 min	36 (29–60)	7 (6–12)	NA
Oscarsson et al. (39)	2–2.4 ATA	90 min	NA	5 times per week	1 time per day	NA	36 (28–40)	7 (6–8)	99.57%
Andren and Bennett (40)	2–2.4 ATA	90 min	5 min	5 times per week	1 time per day	NA	30 (20–50)	6 (4–10)	97.44%
Ouaïssi et al. (41)	2.5 ATA	60 min	NA	5–7 times per week	1 time per day	NA	35 (6–90)	7 (1–18)	98.41%
Velure et al. (42)	2.4 ATA	90 min	NA	5 times per week	1 time per day	NA	30	6	100%
Gaio-Lima et al. (43)	2.4 ATA	NA	NA	5 times per week	1 time per day	90 min	52 (18–70)	10 (4–14)	NA
Woo et al. (35)	2.0 ATA	NA	NA	6 times per week	1 time per day	105 min	24 (12–40)	4 (2–7)	NA
Marshall et al. (36)	2.36 ATA	90 min	NA	NA	1 time per day	NA	30	NA	NA
Hampson et al. (45)	2.36 ATA	90	NA	5 times per week	1 time per day	120 min	42 (26–60)	9 (5–12)	NA
Zhang et al. (46)	2.4 ATA	NA	NA	5–7 times per week	1 time per day	90 min	28	4	NA

ATA, atmosphere absolute; min, minute; NA, no available.

#### 3.7.1 Atmospheric pressure

The pressure parameters are key factors in the therapeutic effect of HBOT. According to the analysis of the included clinical studies, the treatment pressure typically ranges from 2.0 to 2.5 ATA, with 2.4 ATA being the most commonly used pressure level, followed by 2.0 ATA. In a clinical trial that included 27 patients with RE (44), participants received 2.4 ATA hyperbaric oxygen therapy after failure of drug or endoscopic treatment. The results showed that approximately 67% of the participants experienced improvements in symptoms such as pain, rectal bleeding, and tenesmus. Another RCT (20) that included 92 participants found that compared to conventional treatment, 2.0 ATA HBOT significantly reduced the incidence of acute RE, particularly the incidence of RE at RTOG/EORTC grade 3 or higher. Furthermore, at the 12-months follow-up, participants who received HBOT showed better improvement in the Vienna Endoscopy Score compared to the conventional treatment group.

#### 3.7.2 Treatment duration and frequency

All studies utilized 100% pure oxygen. Most clinical trials employed a treatment duration ranging from 90 to 120 min per session, with variations in the duration of pure oxygen inhalation during each session. The majority of studies administered 90 min of pure oxygen inhalation, while other studies utilized 40, 60, or 70 min of inhalation. During the pure oxygen inhalation, participants were given intermittent rest periods of 5–10 min, during which they inhaled air inside the hyperbaric chamber. The treatment frequency typically ranges from 5 to 7 sessions per week, with one session per day. Among these, five sessions per week, with one session per day, was the most commonly adopted treatment frequency in clinical practice.

#### 3.7.3 Total number of HBOT sessions and treatment course

The results of the studies indicated that the total number of treatment sessions typically ranged from 30 to 40, with some studies (41) involving patients receiving up to 90 sessions of HBOT. Based on the total number of treatments and their frequency, the treatment duration typically lasted between 4 and 12 weeks. The determination of the number of treatments and treatment duration needs to take into account the severity of symptoms, patient response, and cost-effectiveness. One study (42) reported that 95 patients with RE who received a total of 30 sessions of HBOT over 6 weeks exhibited significant improvement in gastrointestinal symptoms and quality of life at the 6-months follow-up.

#### 3.7.4 HBOT combined with other treatments

Hyperbaric oxygen therapy was often combined with other treatment modalities, including dietary management characterized by low cellulose, low fat, high calorie, and high protein intake. Additionally, non-steroidal anti-inflammatory drugs (NSAIDs), corticosteroids, probiotics, and mucosal protectants were commonly employed as adjunctive therapies. A RCT (21) demonstrated that the combination of HBOT and mesalazine was more effective than monotherapy. Compared to the monotherapy group, the combination therapy group exhibited significant improvement in lesions, including mucosal erosion, vascular dilation, and ulcerative bleeding, as observed on endoscopy. Additionally, patients in the combination therapy group showed significant improvement in symptoms, including emotional well-being, pain, insomnia, constipation, and diarrhea, as measured by the QLQ-C30 scale. The synergistic effect of combination therapy not only enhances treatment efficacy and shortens recovery time but also improves the overall prognosis of patients. However, when designing combination therapy regimens, it is imperative to consider the mechanisms of action of different treatments and their potential interactions.

#### 3.7.5 Adherence

Adherence plays a crucial role in the clinical implementation of HBOT, as it directly impacts the feasibility and efficacy of the treatment. In the seven clinical trials (22, 29, 38–42) that reported adherence, rates consistently exceeded 90%, indicating high feasibility. However, given the small number of studies and sparse reporting, these findings warrant cautious interpretation.

### 3.8 Safety analysis

The study found that ear barotrauma was the most common side effect, with patients frequently reporting symptoms such as tinnitus and ear pain. Ear barotrauma was usually limited to the eardrum, occasionally affecting the middle ear, and rarely involving the inner ear. Most cases recover on their own during subsequent treatments through enhanced education, accompaniment by family members or medical staff during sessions, adjustments to the pressurization rate, pauses in pressurization, brief decompression, or swallowing and chewing food (22, 31, 34–36, 40, 41, 44, 45). However, some studies (34) have reported a small number of patients with tympanic membrane damage and middle ear effusion. In addition, transient myopia was another common side effect, usually related to the oxidation of lens proteins. It typically resolved on its own within a few weeks after treatment (22, 34–36, 40, 44, 45). No long-term vision impairment had been reported in the studies so far, but close monitoring was still required for elderly patients and those with a history of ophthalmic conditions. Some studies (34, 45) reported that patients exhibited varying degrees of claustrophobia. Multi-person chambers and appropriate psychological counseling could effectively alleviate this issue. For patients with severe claustrophobia, alternative treatment options may need to be considered. It is worth mentioning that some studies (36, 44, 45) reported that a small number of patients developed central nervous system oxygen toxicity during treatment, leading to seizures. However, after symptomatic treatment, these patients successfully completed subsequent therapy without experiencing similar events. Some patients in the studies experienced chest discomfort (35). Therefore, for patients with a history of pulmonary or cardiovascular diseases, such as COPD, hypertension, or heart failure, we recommend conducting a detailed pulmonary or cardiovascular assessment. If necessary, treatment parameters should be adjusted, and individualized treatment should be provided to minimize risks. No studies have reported an association between HBOT and cancer metastasis or recurrence. However, during the initial phase of treatment, HBOT may increase bleeding in patients with RE. One study (38) reported that six patients with proctitis and four patients with cystitis experienced increased bleeding at the beginning of HBOT. One of the proctitis patients had a limited episode of mild rectal bleeding, which was not known before starting HBOT.

Current evidence indicates that the short-term safety of HBOT for RE is generally acceptable. However, the potential for serious adverse events and the long-term risks of tumor recurrence or metastasis necessitate confirmation through further high-quality studies.

## 4 Discussion

Through a meta-analysis of the included RCT studies, our findings indicated that HBOT had a significant advantage in reducing the incidence and severity of RE. It also effectively improved gastrointestinal symptoms, alleviated inflammation, and enhanced immune function. Additionally, the clinical effectiveness of HBOT is closely related to specific treatment parameters. Notably, when used in combination with other treatment modalities, HBOT can further enhance clinical outcomes.

Radiation enteritis results from a combination of various pathophysiological processes, including the activation of the coagulation system, inflammatory responses, epithelial cell regeneration, and collagen deposition (47). In the early stages, ionizing radiation induces oxidative stress via oxygen free radicals, damaging DNA strands and triggering cell apoptosis, thereby damaging the intestinal mucosa (48). Simultaneously, radiation activates abnormal inflammatory responses, increases microvascular permeability, promotes microthrombosis, alters the intestinal wall microenvironment, and damages the vascular endothelium, thereby exacerbating mucosal injury (49, 50). As the disease progresses, sustained radiation damage and delayed effects lead to thinning of the intestinal wall, increased permeability, and the promotion of endothelial cell proliferation, thrombosis, and intestinal fibrosis (51). Immune dysregulation and intestinal microbiota imbalance further intensify the inflammation, complicating the treatment of the condition.

Our findings were consistent with prior research (52), further confirming that HBOT significantly improved clinical symptoms in patients with RE and reduced the incidence and severity of the disease. Mechanistically, HBOT elevates tissue oxygen tension, activates the Nrf2/HO-1 signaling pathway (53), downregulates proinflammatory cytokines such as TNF-α and IL-1β (54), and induces macrophage polarization toward the reparative M2 phenotype (55), thereby reducing the inflammatory burden and stabilizing the local microenvironment. Concurrently, HBOT corrects crypt hypoxia, promotes intestinal stem cell proliferation (56), and upregulates the expression of vascular endothelial growth factor (VEGF) (53, 57) and fibroblast growth factor (FGF) (58), which synergistically drive functional angiogenesis and epithelial regeneration, improve perfusion, and strengthen barrier function. These anti-inflammatory and reparative processes were mutually reinforcing: the reduction in inflammation facilitated barrier reconstruction and reperfusion, which in turn further suppressed inflammation. Thus, HBOT helps disrupt the vicious cycle of endothelial injury–ischemia–inflammation–fibrosis in RE, promoting restoration of mucosal structure and function.

Our findings indicate that the treatment pressure in clinical HBOT typically ranges between 2.0 and 2.5 ATA. The relationship between treatment pressure parameters and tissue oxygen partial pressure is closely linked, influencing the oxygen gradient between capillaries and tissues. Both 2.0 ATA and 2.4 ATA are the most commonly used treatment pressures in clinical practice. Under both pressure parameters, HBOT has achieved satisfactory therapeutic outcomes. One study (59) reported that after HBOT at 2.4 ATA, there was a reduction in prostaglandin E2 production, decreased activity of myeloperoxidase and nitric oxide synthase, and improvements in tissue edema and inflammatory response. In contrast, 2.0 ATA stabilized and activated hypoxia-inducible factor-1 (60), reducing levels of transforming growth factor-β 1 (29), which helped inhibit intestinal tissue fibrosis and promote wound healing. Currently, there is insufficient evidence to assess the efficacy differences between the two treatment pressures. However, a large retrospective analysis (61) found no significant difference between these two HBOT pressure levels (2.0 ATA vs. 2.4 ATA) in the treatment of radiation cystitis. It is worth noting that increased pressure may raise the risk of central nervous system oxygen toxicity (CNS-OT). CNS-OT is a rare but dangerous side effect of HBOT, characterized by seizures, with an incidence rate ranging between 1:10,000 and 1:50,000 (62). This study found that some research reports indicated that patients with RE experienced seizures after receiving HBOT at 2.36 ATA (36, 45) or 2.4 ATA (44). Although these patients successfully completed subsequent treatments after timely symptomatic management, with no recurrence or more severe consequences, we believe that to minimize potential risks, HBOT at 2.0 ATA may offer relatively more benefits while ensuring clinical efficacy.

The effectiveness of treatment depends not only on the treatment pressure parameters but also on the selection of treatment duration, frequency, and total course. Currently, the commonly used clinical protocol is 90–120 min per session, five sessions per week, one session per day, with a total of 30–40 sessions. Some scholars suggested that for radiation-induced tissue damage, a treatment duration of at least 90 min per session and a total of at least 40 sessions were necessary to achieve better clinical outcomes (63). However, a study found that approximately 45% of patients who did not respond to treatment were diagnosed with local tumor recurrence. To further reduce the risk of tumor recurrence, it is recommended that patients with RE not initially undergo more than 40 sessions of HBOT. Additionally, if symptoms show little or no improvement, cancer recurrence should be evaluated before any further HBOT (34). Additionally, the severity of the patient’s condition is an important factor in adjusting treatment duration and course. A clinical study (40) found that after receiving a total of 30 sessions (ranging from 20 to 50), over 4 to 10 weeks of HBOT, patients with RE showed a significant reduction in their LENT-SOMA scores and improvement in clinical symptoms. However, the decrease in LENT-SOMA scores was not associated with an increase in the number of HBOT sessions. The study also noted that some severe cases might still benefit from extended treatment. For patients with significant symptom improvement, the number of subsequent treatments can be appropriately reduced; whereas for patients with poor responses, extending the treatment course or increasing treatment frequency should be considered.

Age and radiation dose are also critical factors influencing treatment efficacy (64). Studies have shown that with increasing age and higher radiation doses, the incidence and severity of radiation enteritis (RE) correspondingly increase (65, 66). High-dose radiation can cause more severe microvascular injury and apoptosis (66), potentially limiting HBOT’s capacity to induce angiogenesis and epithelial regeneration. Although a retrospective cohort study involving 88 patients with RE showed no significant association between age and response to HBOT (19), older patients may require longer treatment durations due to decreased tissue repair capacity and the presence of comorbidities (67). Therefore, in clinical research and practice, radiation dose and age should be treated as core stratification factors to more precisely estimate the expected benefit of HBOT and develop individualized treatment strategies.

### 4.1 Strengths and limitations

To our knowledge, this is currently the largest systematic review in clinical research, involving 22 clinical studies and 1,318 RE participants. The study not only confirms that HBOT can effectively improve patients’ gastrointestinal symptoms, promote intestinal mucosal repair, reduce inflammation, and enhance immune function, but also highlights its significant advantage in reducing the incidence of RE, especially in lowering the rate of severe cases. Additionally, the study provides an in-depth analysis of the clinical parameters of HBOT, further exploring its potential mechanisms of action and offering valuable evidence-based support for its clinical application in treating RE. This study also has certain limitations. First, the number of included RCTs was limited, sample sizes were small, and some NRITs carried a high risk of bias, which constrained our ability to further explore differences in HBOT efficacy across parameter settings and reduced the overall quality of the evidence. Second, several RCTs did not clearly report randomization methods or blinding, potentially introducing placebo effects and observer bias. Many NRITs did not adequately account for confounders such as age, tumor type, and radiation dose, nor did they perform multivariable analyses, thereby reducing the reliability of the findings. Third, because the number of included RCTs was small, we were unable to assess publication bias. Moreover, since most RCTs were conducted in China, regional bias may exist, and applicability to other regions requires further validation. Fourth, the limited number of RCTs precluded subgroup analyses or meta-regression to investigate the sources of high heterogeneity observed in some outcomes. Fifth, there was a lack of health-related quality of life (HRQoL) and economic evidence, preventing a rigorous assessment of HBOT’s long-term impact on patients’ quality of life and cost-effectiveness.

### 4.2 Outlook

Despite the potential benefits of HBOT for treating RE, the current evidence remains limited due to a lack of high-quality randomized controlled trials. There is an urgent need for large-scale, multicenter RCTs to rigorously evaluate different combinations of HBOT parameters, including pressure levels, treatment duration, and frequency, on clinical efficacy and prognosis. Additionally, future studies should prioritize systematic assessments of HRQoL and economic impact. This will require the prespecification of validated HRQoL instruments, extending follow-up periods, and conducting formal cost-effectiveness analyses to robustly determine HBOT’s effects on long-term patient well-being and resource utilization. Equally important is the development of personalized treatment strategies. Creating AI-assisted decision-support systems represents a promising approach to optimizing HBOT delivery. Such systems should integrate multimodal patient data, including age, comorbidities, radiation exposure history, and genomic markers, to guide individualized parameter selection and enhance treatment precision.

## 5 Conclusion

This study found that HBOT is safe and effective for improving gastrointestinal symptoms, promoting intestinal mucosal repair, reducing inflammation, and enhancing immune function in patients with RE. Notably, HBOT also significantly reduces the incidence of RE, especially in lowering the rate of severe cases. Commonly reported clinical parameters include five sessions per week, lasting 90–120 min each, for a total of 30–40 sessions at 2.0–2.5 ATA. However, these parameters are derived from existing literature, and the evidence remains insufficient to establish an optimal regimen. Therefore, despite the potential benefits of HBOT for RE, clinical use should be approached with caution, incorporating individualized adjustments to treatment parameters and close monitoring of patient responses and potential adverse effects.

## Data Availability

The original contributions presented in this study are included in this article/Supplementary material, further inquiries can be directed to the corresponding authors.
