# Supplementary material for: Hyperbaric oxygen therapy for radiation enteritis and clinical parameters: a systematic review and meta-analysis
Source: Front Med (Lausanne). 2025 Oct 10;12:1632414. doi: 10.3389/fmed.2025.1632414 (PMC12549627; doi:10.3389/fmed.2025.1632414)
Supplement: Supplementary file 1 [file Data_Sheet_1.docx]

# Hyperbaric Oxygen Therapy for Radiation Enteritis and Clinical Parameters: A Systematic Review and Meta-Analysis

**Yuhan Wang^1^**^†^**, Kunlin Kuang^1^**^†^**, Xuhui Yang^1^**^†^**, Zili Tang^1^, Minjiao Jiang^2^, Ling Zhao^1,3,4＊^**

**Supplement Table**

**Supplement Table 1 Pubmed **search strategy****

| **NO.** | **Search Items** |
| --- | --- |
| **#1** | Neoplasm[MeSH] |
| **#2** | ([Carcinoma](http://www.baidu.com/link?url=eGtZz9YjBs3b90XYeCcCWLsh_Ilkwi8knZ801q4vPwQgbHynFTPLw85S730p6dgNx3NlIe4xMn-GJFdVGCOXcmsCfGsbeXSsqx-j3GLmS6oK1xB9nWUshrC3lhTCgfkB) OR OR Cancer OR Tumor OR Malignancy)[Title/Abstract] |
| **#3** | #1 OR #2 |
| **#4** | Hyperbaric oxygen[MeSH] |
| **#5** | (Hyperbaric oxygen therapy)[Title/Abstract] |
| **#6** | #4 OR #5 |
| **#7** | Radiotherapy[MeSH] |
| **#8** | ([Brachytherapy](https://www.ncbi.nlm.nih.gov/mesh/68001918) OR [Neoadjuvant Therapy](https://www.ncbi.nlm.nih.gov/mesh/68020360) OR [Lymphatic Irradiation](https://www.ncbi.nlm.nih.gov/mesh/68015182) OR Radiation)[Title/Abstract] |
| **#9** | (Radiation enteritis OR Radiation colitis OR Radiation proctitis OR Diarrhea OR [hemafecia](https://fanyi.so.com/?src=onebox#haemafecia) OR [hematochezia](https://fanyi.so.com/?src=onebox#%20haematochezia) OR Gastrointestinal Hemorrhages OR bowel dysfunction OR [Intestinal Diseases](https://www.ncbi.nlm.nih.gov/mesh/68007410) OR Bowel function)[Title/Abstract] |
| **#10** | #7 OR #8 OR #9 |
| **#11** | Clinical OR Trial [Title/Abstract] |
| **#12** | #3 AND #6 AND #10 AND #11 |

**Supplement Table 2 Web of Science **search strategy****

| **NO.** | **Search Items** |
| --- | --- |
| **#1** | TS=(Neoplasm) |
| **#2** | AB=([Carcinoma](http://www.baidu.com/link?url=eGtZz9YjBs3b90XYeCcCWLsh_Ilkwi8knZ801q4vPwQgbHynFTPLw85S730p6dgNx3NlIe4xMn-GJFdVGCOXcmsCfGsbeXSsqx-j3GLmS6oK1xB9nWUshrC3lhTCgfkB) OR OR Cancer OR Tumor OR Malignancy) |
| **#3** | #1 OR #2 |
| **#4** | TS=(Hyperbaric oxygen) |
| **#5** | AB=(Hyperbaric oxygen therapy) |
| **#6** | #4 OR #5 |
| **#7** | TS=(Radiotherapy) |
| **#8** | AB=([Brachytherapy](https://www.ncbi.nlm.nih.gov/mesh/68001918) OR [Neoadjuvant Therapy](https://www.ncbi.nlm.nih.gov/mesh/68020360) OR [Lymphatic Irradiation](https://www.ncbi.nlm.nih.gov/mesh/68015182) OR Radiation) |
| **#9** | AB=(Radiation enteritis OR Radiation colitis OR Radiation proctitis OR Diarrhea OR [hemafecia](https://fanyi.so.com/?src=onebox#haemafecia) OR [hematochezia](https://fanyi.so.com/?src=onebox#%20haematochezia) OR Gastrointestinal Hemorrhages OR bowel dysfunction OR [Intestinal Diseases](https://www.ncbi.nlm.nih.gov/mesh/68007410) OR Bowel function) |
| **#10** | #7 OR #8 OR #9 |
| **#11** | AB=(Clinical OR Trialy) |
| **#12** | #3 AND #6 AND #10 AND #11 |

**Supplement Table 3 Embase **search strategy****

| **NO.** | **Search Items** |
| --- | --- |
| **#1** | 'Neoplasm'/exp |
| **#2** | ([Carcinoma](http://www.baidu.com/link?url=eGtZz9YjBs3b90XYeCcCWLsh_Ilkwi8knZ801q4vPwQgbHynFTPLw85S730p6dgNx3NlIe4xMn-GJFdVGCOXcmsCfGsbeXSsqx-j3GLmS6oK1xB9nWUshrC3lhTCgfkB) OR OR Cancer OR Tumor OR Malignancy):ab,kw,ti |
| **#3** | #1 OR #2 |
| **#4** | 'Hyperbaric oxygen'/exp |
| **#5** | (Hyperbaric oxygen therapy):ab,kw,ti |
| **#6** | #4 OR #5 |
| **#7** | 'Radiotherapy'/exp |
| **#8** | ([Brachytherapy](https://www.ncbi.nlm.nih.gov/mesh/68001918) OR [Neoadjuvant Therapy](https://www.ncbi.nlm.nih.gov/mesh/68020360) OR [Lymphatic Irradiation](https://www.ncbi.nlm.nih.gov/mesh/68015182) OR Radiation):ab,kw,ti |
| **#9** | (Radiation enteritis OR Radiation colitis OR Radiation proctitis OR Diarrhea OR [hemafecia](https://fanyi.so.com/?src=onebox#haemafecia) OR [hematochezia](https://fanyi.so.com/?src=onebox#%20haematochezia) OR Gastrointestinal Hemorrhages OR bowel dysfunction OR [Intestinal Diseases](https://www.ncbi.nlm.nih.gov/mesh/68007410) OR Bowel function):ab,kw,ti |
| **#10** | #7 OR #8 OR #9 |
| **#11** | (Clinical OR Trialy):ab,kw,ti |
| **#12** | #3 AND #6 AND #10 AND #11 |

**Supplement Table 4 Cochrane Library **search strategy****

| **NO.** | **Search Items** |
| --- | --- |
| **#1** | MeSH descriptor: [Neoplasm] explode all trees |
| **#2** | ([Carcinoma](http://www.baidu.com/link?url=eGtZz9YjBs3b90XYeCcCWLsh_Ilkwi8knZ801q4vPwQgbHynFTPLw85S730p6dgNx3NlIe4xMn-GJFdVGCOXcmsCfGsbeXSsqx-j3GLmS6oK1xB9nWUshrC3lhTCgfkB) OR OR Cancer OR Tumor OR Malignancy):ti,ab,kw |
| **#3** | #1 OR #2 |
| **#4** | MeSH descriptor: [Hyperbaric oxygen] explode all trees |
| **#5** | (Hyperbaric oxygen therapy):ti,ab,kw |
| **#6** | #4 OR #5 |
| **#7** | MeSH descriptor: [Radiotherapy] explode all trees |
| **#8** | ([Brachytherapy](https://www.ncbi.nlm.nih.gov/mesh/68001918) OR [Neoadjuvant Therapy](https://www.ncbi.nlm.nih.gov/mesh/68020360) OR [Lymphatic Irradiation](https://www.ncbi.nlm.nih.gov/mesh/68015182) OR Radiation):ti,ab,kw |
| **#9** | (Radiation enteritis OR Radiation colitis OR Radiation proctitis OR Diarrhea OR [hemafecia](https://fanyi.so.com/?src=onebox#haemafecia) OR [hematochezia](https://fanyi.so.com/?src=onebox#%20haematochezia) OR Gastrointestinal Hemorrhages OR bowel dysfunction OR [Intestinal Diseases](https://www.ncbi.nlm.nih.gov/mesh/68007410) OR Bowel function):ti,ab,kw |
| **#10** | #7 OR #8 OR #9 |
| **#11** | (Clinical OR Trialy):ti,ab,kw |
| **#12** | #3 AND #6 AND #10 AND #11 |

**Supplement Table 5 Wiley **search strategy****

| **NO.** | **Search Items** |
| --- | --- |
| **#1** | Neoplasm[Title] |
| **#2** | ([Carcinoma](http://www.baidu.com/link?url=eGtZz9YjBs3b90XYeCcCWLsh_Ilkwi8knZ801q4vPwQgbHynFTPLw85S730p6dgNx3NlIe4xMn-GJFdVGCOXcmsCfGsbeXSsqx-j3GLmS6oK1xB9nWUshrC3lhTCgfkB) OR OR Cancer OR Tumor OR Malignancy)[Abstract] |
| **#3** | #1 OR #2 |
| **#4** | Hyperbaric oxygen[Title] |
| **#5** | (Hyperbaric oxygen therapy)[Abstract] |
| **#6** | #4 OR #5 |
| **#7** | Radiotherapy[Title] |
| **#8** | ([Brachytherapy](https://www.ncbi.nlm.nih.gov/mesh/68001918) OR [Neoadjuvant Therapy](https://www.ncbi.nlm.nih.gov/mesh/68020360) OR [Lymphatic Irradiation](https://www.ncbi.nlm.nih.gov/mesh/68015182) OR Radiation)[Abstract] |
| **#9** | (Radiation enteritis OR Radiation colitis OR Radiation proctitis OR Diarrhea OR [hemafecia](https://fanyi.so.com/?src=onebox#haemafecia) OR [hematochezia](https://fanyi.so.com/?src=onebox#%20haematochezia) OR Gastrointestinal Hemorrhages OR bowel dysfunction OR [Intestinal Diseases](https://www.ncbi.nlm.nih.gov/mesh/68007410) OR Bowel function)[Abstract] |
| **#10** | #7 OR #8 OR #9 |
| **#11** | Clinical OR Trial [Abstract] |
| **#12** | #3 AND #6 AND #10 AND #11 |

**Supplement Table 6 CNKI **search strategy****

| ****search strategy**** |
| --- |
| (TKA=(‘高压氧治疗’) OR SU=(‘高压氧’)) AND (TKA=(‘放疗’+‘放射性治疗’+‘辐射’) AND (TKA=(‘肠炎’+‘肠病’+‘肠溃疡’+‘直肠炎’+‘结肠炎’+‘大肠炎’+‘肠粘膜损伤’+‘小肠炎’+‘便血’+‘消化道出血’+‘血便’+‘黑便’+‘腹泻’+‘泄泻’) AND (TKA=(‘临床研究’+‘临床观察’+‘试验’+‘临床’) |

**Supplement Table 7 Wanfang **search strategy****

| ****search strategy**** |
| --- |
| (主题=(高压氧治疗) OR 题名或关键词=(高压氧)) AND (主题=(放疗+放射性治疗+‘辐射) AND (题名或关键词=(肠炎+肠病+肠溃疡+直肠炎+结肠炎+大肠炎+肠粘膜损伤+小肠炎+便血+消化道出血+血便+黑便+腹泻+泄泻) AND (题名或关键词=(临床研究+临床观察+试验+临床) |

**Supplement Table 8 VIP **search strategy****

| ****search strategy**** |
| --- |
| (M=(高压氧治疗 OR 高压氧) AND (M=(放疗+放射性治疗+‘辐射) AND (M=(肠炎+肠病+肠溃疡+直肠炎+结肠炎+大肠炎+肠粘膜损伤+小肠炎+便血+消化道出血+血便+黑便+腹泻+泄泻) AND (M=(临床研究+临床观察+试验+临床) |

**Supplement Table 9 **SinoMed search strategy****

| ****search strategy**** |
| --- |
| (主题词(高压氧治疗) OR 常用字段：智能:(高压氧)) AND (主题词(放疗+放射性治疗+‘辐射) AND (常用字段：智能:(肠炎+肠病+肠溃疡+直肠炎+结肠炎+大肠炎+肠粘膜损伤+小肠炎+便血+消化道出血+血便+黑便+腹泻+泄泻) AND (常用字段：智能:(临床研究+临床观察+试验+临床) |

**Supplementary Table 10 Summary of GRADE recommendations**

| Outcome | NO.of studies | NO.of patients | Quality of assessmet | | | | | Quality of evidence |
| --- | --- | --- | --- | --- | --- | --- | --- | --- |
|  |  |  | Risk of bias | Inconsistency | Indirectness | Imprecision | Other considerations |  |
| Incidence of radiation enteritis | 2 | 138 | Serious^1^ | None | None | None | Undetected | Moderate |
| **Incidence of severe cases** | 2 | 178 | Serious^1^ | None | None | None | Undetected | Moderate |
| LENT-SOMA | 2 | 121 | Serious^1^ | None | None | None | Undetected | Moderate |
| TNF-α | 2 | 172 | Serious^1^ | serious^2^ | None | None | Undetected | Low |
| CRP | 2 | 172 | Serious^1^ | serious^2^ | None | None | Undetected | Low |
| IL-6 | 2 | 172 | Serious^1^ | serious^2^ | None | None | Undetected | Low |
| IgA | 2 | 172 | Serious^1^ | serious^2^ | None | None | Undetected | Low |
| IgM | 2 | 172 | Serious^1^ | serious^2^ | None | None | Undetected | Low |
| IgG | 2 | 172 | Serious^1^ | serious^2^ | None | None | Undetected | Low |

Supplement: 1: High risk of bias; 2: High heterogeneity

**Supplement Table 11 Results of risk of bias assessment in ROBINS-I tool**

| Author | Bias due to confounding | Bias in selection of participants into the study | Bias in classification of interventions | Bias due to deviations from intended interventions | Bias due to missing data | Bias in measurement of outcomes | Bias in selection of the reported result | Overall level |
| --- | --- | --- | --- | --- | --- | --- | --- | --- |
| Li2006^[38]^ | Low risk of bias | Low risk of bias | Low risk of bias | Low risk of bias | Low risk of bias | Low risk of bias | Low risk of bias | Low risk of bias |
| Monteiro2023^[20]^ | Low risk of bias | Serious risk of bias: Inconsistent intervention and follow-up duration among some participants. | Low risk of bias | Low risk of bias | Low risk of bias | Low risk of bias | Low risk of bias | Serious risk of bias |
| Mayer2001^[39]^ | Critical risk of bias: The study did not adjust for important confounding factors such as age, gender, and the degree of tissue damage, and only performed univariate analysis. | Serious risk of bias: Inconsistent intervention and follow-up duration among some participants. | Low risk of bias | Low risk of bias | Low risk of bias | Low risk of bias | Low risk of bias | Critical risk of bias |
| Dall'Era2006^[45]^ | Critical risk of bias: The study did not adjust for important confounding factors such as age, gender, and the degree of tissue damage, and only performed univariate analysis. | Serious risk of bias: Inconsistent intervention and follow-up duration among some participants. | Low risk of bias | Low risk of bias | Low risk of bias | Moderate risk of bias: The study includes subjective outcome measures. | Low risk of bias | Critical risk of bias |
| Oscarsson2013^[40]^ | Critical risk of bias: The study did not adjust for important confounding factors such as age, gender, and the degree of tissue damage, and only performed univariate analysis. | Serious risk of bias: Inconsistent intervention and follow-up duration among some participants. | Low risk of bias | Low risk of bias | Low risk of bias | Low risk of bias | Low risk of bias | Critical risk of bias |
| Andren2020^[41]^ | Critical risk of bias: The study did not adjust for important confounding factors such as age, gender, and the degree of tissue damage, and only performed univariate analysis. | Serious risk of bias: Inconsistent intervention and follow-up duration among some participants. | Low risk of bias | Low risk of bias | Low risk of bias | Low risk of bias | Low risk of bias | Critical risk of bias |
| Ouaïssi2014^[42]^ | Critical risk of bias: The study did not adjust for important confounding factors such as age, gender, and the degree of tissue damage, and only performed univariate analysis. | Serious risk of bias: Inconsistent intervention and follow-up duration among some participants. | Low risk of bias | Low risk of bias | Low risk of bias | Low risk of bias | Low risk of bias | Critical risk of bias |
| Velure2022^[43]^ | Low risk of bias | Low risk of bias | Low risk of bias | Low risk of bias | Low risk of bias | Low risk of bias | Low risk of bias | Low risk of bias |
| Gaio-Lima2022^[44]^ | Critical risk of bias: The study did not adjust for important confounding factors such as age, gender, and the degree of tissue damage, and only performed univariate analysis. | Low risk of bias | Low risk of bias | Low risk of bias | Moderate risk of bias: Among the 112 participants, 24 were lost to follow-up. | Low risk of bias | Low risk of bias | Critical risk of bias |
| Alvaro-Villegas2011^[33]^ | Low risk of bias | Low risk of bias | Moderate risk of bias: Participants were assigned to groups based on the available medical resources at the time of referral. | Moderate risk of bias: The two treatment groups received interventions with different frequencies and durations. | Low risk of bias | Low risk of bias | Low risk of bias | Moderate risk of bias |
| Woo1997^[36]^ | Critical risk of bias: The study did not adjust for important confounding factors such as age, gender, and the degree of tissue damage, and only performed univariate analysis. | Serious risk of bias: Inconsistent intervention and follow-up duration among some participants. | Low risk of bias | Low risk of bias | Low risk of bias | Moderate risk of bias: The study includes subjective outcome measures. | Low risk of bias | Critical risk of bias |
| Marshall2007^[37]^ | Critical risk of bias: The study did not adjust for important confounding factors such as age, gender, and the degree of tissue damage, and only performed univariate analysis. | Serious risk of bias: Inconsistent intervention and follow-up duration among some participants. | Low risk of bias | Low risk of bias | Low risk of bias | Low risk of bias | Low risk of bias | Critical risk of bias |
| [Hampson2012](https://pubmed.ncbi.nlm.nih.gov/?size=200&term=Hampson+NB&cauthor_id=22139864)^[46]^ | Critical risk of bias: The study did not adjust for important confounding factors such as age, gender, and the degree of tissue damage, and only performed univariate analysis. | Serious risk of bias: Inconsistent intervention and follow-up duration among some participants. | Low risk of bias | Low risk of bias | Low risk of bias | Moderate risk of bias: The study includes subjective outcome measures. | Low risk of bias | Critical risk of bias |
| Zhang2013^[47]^ | Low risk of bias | Low risk of bias | Moderate risk of bias: Participants were assigned to groups based on the available medical resources at the time of referral. | Low risk of bias | Low risk of bias | Moderate risk of bias: The study includes subjective outcome measures. | Low risk of bias | Moderate risk of bias |
